# MicroRNA125b-mediated Hedgehog signaling influences liver regeneration by chorionic plate-derived mesenchymal stem cells

**DOI:** 10.1038/srep14135

**Published:** 2015-09-15

**Authors:** Jeongeun Hyun, Sihyung Wang, Jieun Kim, Gi Jin Kim, Youngmi Jung

**Affiliations:** 1 Department of Integrated Biological Science, Pusan, 609-735, Korea; 2 Department of Biological Sciences, College of Natural Science, Pusan National University, Pusan, 609-735, Korea; 3 Department of Biomedical Science, CHA University, Seongnam, 463-400, Korea

## Abstract

Although chorionic plate-derived mesenchymal stem cells (CP-MSCs) were shown to promote liver regeneration, the mechanisms underlying the effect remain unclear. Hedgehog (Hh) signaling orchestrates tissue reconstruction in damaged liver. MSCs release microRNAs mediating various cellular responses. Hence, we hypothesized that microRNAs from CP-MSCs regulated Hh signaling, which influenced liver regeneration. Livers were obtained from carbon tetrachloride (CCl_4_)-treated rats transplanted with human CP-MSCs (Tx) or saline (non-Tx). Sonic Hh, one of Hh ligands, increased in CCl_4_-treated liver, whereas it decreased in CP-MSC-treated liver with CCl_4_. The expression of Hh-target genes was significantly downregulated in the Tx. Reduced expansion of progenitors and regressed fibrosis were observed in the liver of the Tx rats. CP-MSCs suppressed the expression of Hh and profibrotic genes in co-cultured LX2 (human hepatic stellate cell) with CP-MSCs. MicroRNA-125b targeting *smo* was retained in exosomes of CP-MSCs. CP-MSCs with microRNA-125b inhibitor failed to attenuate the expression of Hh signaling and profibrotic genes in the activated HSCs. Therefore, these results demonstrated that microRNA-125b from CP-MSCs suppressed the activation of Hh signaling, which promoted the reduced fibrosis, suggesting that microRNA-mediated regulation of Hh signaling contributed to liver regeneration by CP-MSCs.

Liver disease is one of the most common diseases worldwide. Mild liver disease can be cured by appropriate treatments. However, chronic liver disease is characterized by permanent changes to liver and associated with a poor outcome and high mortality. Although liver transplantation is the best option for patients with chronic liver disease, there are several limitations, such as an absence of donors and post-transplant complications, including immune rejection response and death of the donor or recipient in worst-case scenarios^1^. Therefore, stem cell therapy has been heralded as an alternative treatment strategy for patients who suffer from various chronic diseases, including cancer.

Mesenchymal stem cells (MSCs) are multipotent stem cells, which can differentiate into mesenchymal lineages, such as bone, cartilage, muscle and adipose, under specific conditions of culture^2^. MSCs isolated from bone marrow (BM) or cord blood differentiate into hepatocyte-like cells *in vitro*^3^,^4^. A therapeutic effect of MSCs from BM or cord blood on cirrhotic livers was demonstrated in experimental animal models^3^,^5^. Recently, chorionic plate-derived mesenchymal stem cells (CP-MSCs) have been reported as an attractive source for regenerative therapy. CP-MSCs have round-spindle shape of MSCs and express stem cell markers (Oct-4, Nanog, Sox2, and TERT), germ layer markers (NF68, cardiac muscle, α-fectoprotein) and an immunomodulator gene (HLA-G)^6^. They also share common characteristics with BM-MSCs and have several advantages, such as mutipotency, easy accessibility, abundance, and immunosuppressive characteristics^6^,^7^. CP-MSC was shown to differentiate into osteogenic, adipogenic, chondrogenic and hepatogenic lineages under certain conditions, *in vitro*^6^. Studies have shown that the transplanted human CP-MSCs reduce fibrosis in lung and liver of murine models^6^,^8^. However, it remains unclear how CP-MSCs decrease the fibrosis and contribute to liver regeneration. It was considered that MSCs repaired tissues by engrafting and differentiating to replace the damaged tissues, because of their remarkable differentiation potential and homing ability. But, the number of long-term engrafted MSCs was very few in most studies, although engraftment and differentiation of MSCs were reported in some experimental animals with severe tissue damage or with local infusion of large numbers of cells^9^,^10^. Hence, therapeutic effects of MSCs could be explained by cell-to-cell interaction or paracrine control^10^. Emerging evidence reports that MSCs secrete the regulatory factors including cytokines and various forms of transcripts, and are involved in the repair process through intercellular communications based on their paracrine activity^11^.

MicroRNA (miRNA), a endogenous small noncoding RNA of about 22 nucleotides long, controls negatively gene expression via the miRNA-containing RNA-induced silencing complex (miRISC), which binds to complementary sequences within target mRNA and then degrades mRNA or inhibits translation^12^. MiRNAs regulate a wide range of cellular processes in the healthy organism, and dysregulation of miRNAs is closely associated with diseases. Thus, miRNAs are good targets in the diagnosis and treatment of various diseases, including liver fibrosis. Recent studies demonstrated that MSCs retained miRNAs regulating self-renewal and differentiation of MSCs and that these miRNAs differed, depending on the origin of the MSCs^13^,^14^. MSCs release microvesicles (MVs) or exosomes carrying specific miRNAs, and MVs or exosomes deliver these miRNAs to their target cells^15^,^16^. However, the effect of miRNAs released by MSCs on the regeneration process remains poorly understood.

The hedgehog (Hh) pathway plays important roles in tissue remodeling in adults^17^,^18^. Hh ligands, Sonic Hh (Shh), Indian Hh (Ihh), and Desert Hh (Dhh), bind to the Hh receptor, Patched (Ptc), which activates cellular activities of the Smoothened (Smo) receptor via Glioblastomas (Glis). Active Smo translocates the cytoplasmic Gli family (Gli1, Gli2 and Gli3) into the nucleus, and nuclear Glis act as transcriptional factors, activating Hh signaling. In the injured liver, dying hepatocytes were shown to release Hh ligands^19^,^20^. These Hh ligands trigger the proliferation of Hh-responsive cells, such as hepatic progenitor cells and hepatic stellate cells (HSCs), which also produce Hh ligands and amplify the activity of Hh signaling in those cells in an autocrine and paracrine manner^18^,^19^,^20^,^21^. In addition, Hh signaling was shown to induce an epithelial-to-mesenchymal transition (EMT) and to activate quiescent (Q)-HSCs into myofibroblastic (MF)-HSCs^21^,^22^. These findings demonstrate that Hh signaling is critically important in hepatic fibrogenesis.

In a previous study, transplanted human CP-MSCs induced liver regeneration in carbon tetrachloride (CCl_4_)-induced cirrhotic livers of rat^6^. Hh signaling regulates repair response in the damaged liver and MSCs retain regulatory miRNAs. Based on these observations, we hypothesize that CP-MSCs produce miRNAs regulating Hh signaling, which influence the reduced fibrosis, contributing liver regeneration in CP-MSC-transplanted liver. Our results confirmed out hypothesis and demonstrated that miRNA-125b released by CP-MSCs led to the inactivation of Hh signaling, which, in turn, promoted the reduced fibrosis in liver.

## Results

### Distinct expression of the Hh ligands, Sonic and Indian hedgehog, in the repair process in liver

**Figure 1:**
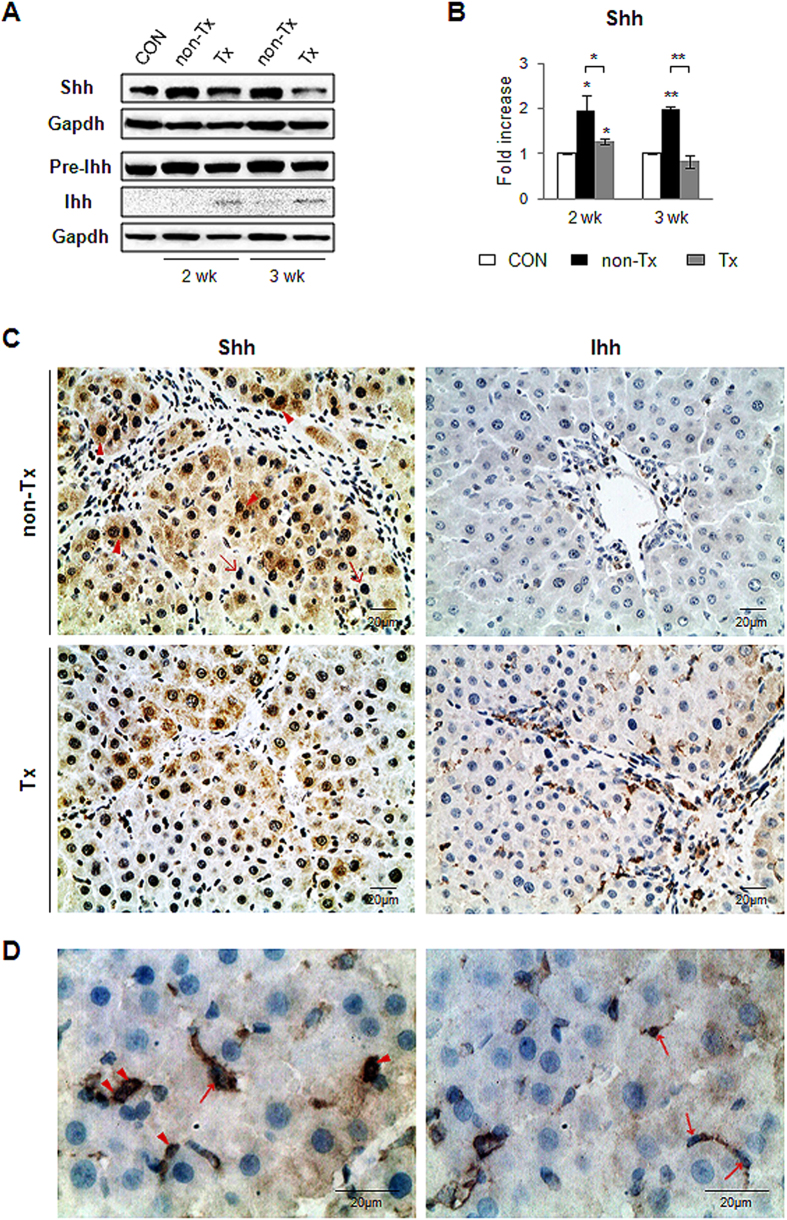
Hh ligands, Shh and Ihh, are differentially expressed in liver of Tx rats. (**A**,**B**) Western blot analysis for Shh (27 kDa), Ihh (19 kDa: processed form/42 kDa: precursor form) and Gapdh (36 kDa, as an internal control). Data shown represent one of three experiments with similar results ((**A**) immunoblot/(**B**) band intensity). The mean ± SD results obtained by measuring the band density of three different blots are graphed (*p < 0.05, **p < 0.005 vs. control). (**C**) Immunohistochemistry for Shh and Ihh in liver sections from representative non-Tx and Tx rats at two weeks post transplantation (×40). Shh-positive and -negative hepatocytes are indicated by arrowhead and arrow, respectively. (**D**) Magnified image of Ihh-positive hepatic oval- and stellate-looking cells indicated by arrowhead and arrow, respectively (×100).

The expression of Hh signaling pathway was known to increase with severity of liver damage and fibrosis^18^, and contribute to liver reconstitution after damages^17^,^23^. Hence, we examined the expression of Hh molecules in the liver with CP-MSC transplantation. The expression of Shh, one of Hh ligands, was lower in the Tx group than in the non-Tx group at two weeks and gradually decreased to baseline Shh expression at three weeks, as assessed by western blot (Fig. 1A,B). Immunostaining for Shh in the liver specimens from the non-Tx rats demonstrated that Shh was expressed in the periportal hepatocytes and that its expression was robust immediately adjacent to fibrotic tracts at both two and three weeks (Fig. 1C, Supplementary Fig. S1). These Shh-expressing hepatocytes (indicated by an arrowhead) were also detected at two weeks, but significantly less in the Tx group than in the non-Tx group. Those cells were rarely detected in the livers from the Tx group at three weeks. Interestingly, we found a dramatic difference in the expression of Ihh, another Hh ligand, and Shh in response to CP-MSC transplantation. The expression level of Ihh protein was upregulated in the Tx rats compared to the non-Tx rats (Fig. 1A). The Tx rats contained a larger quantity of the activated form (19 kDa) and a smaller quantity of the precursor (42 kDa) of Ihh than the non-Tx rats. Ihh-positive cells in the liver from the Tx rats were predominantly located in the portal tract areas and expressed by HSCs and oval-looking cells (indicated by an arrow and an arrow head, respectively) but not hepatocytic cells (Fig. 1C,D, Supplementary Fig. S1), whereas these cells were rarely detected in the livers from the non-Tx rats. These results indicate that Shh and Ihh might have distinct effects on CP-MSC-mediated liver regeneration.

### Decreased expression of Hh-target genes, Smo, Gli2 and Gli3, in the livers of the Tx group

**Figure 2:**
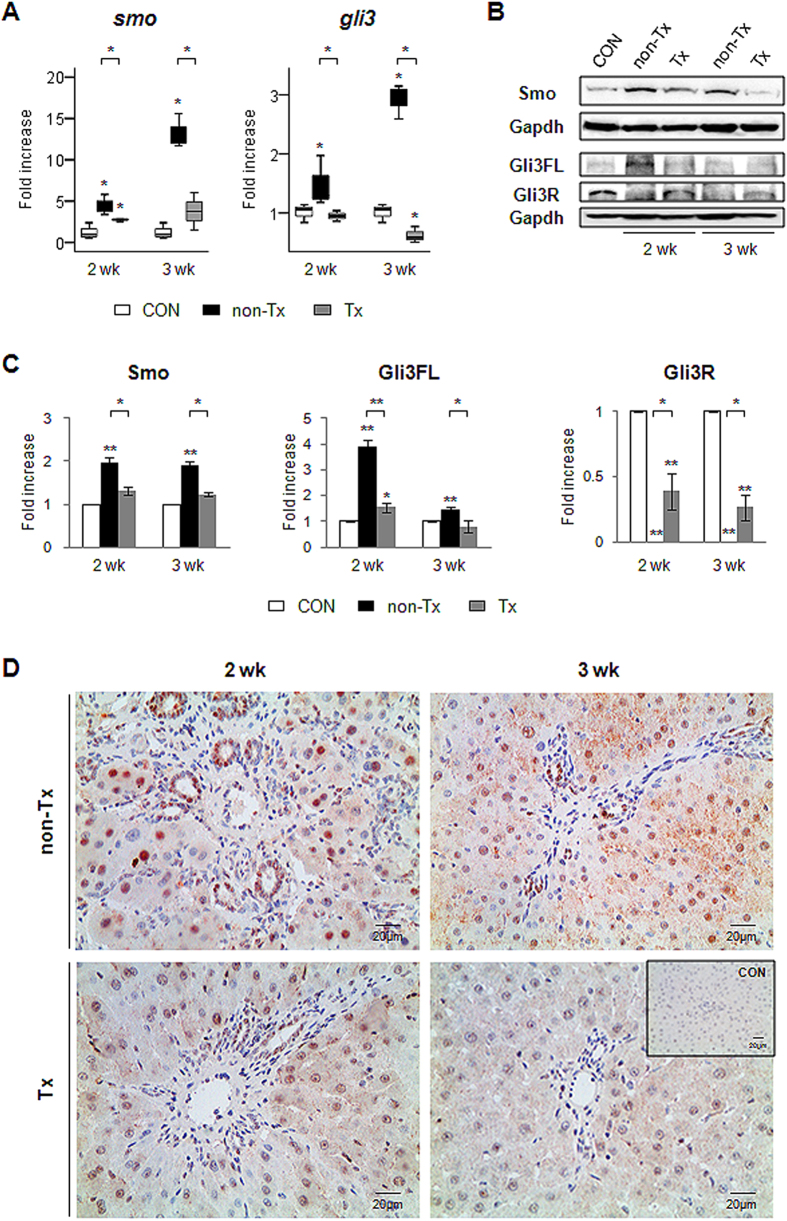
Downregulation of Hh activator, *Smo*, and Hh target gene, *Gli3*, in the CP-MSC-transplanted livers. (**A**) QRT-PCR of *smo* and *gli3* in the livers from the healthy (CON), non-Tx and Tx group. Medians and ranges of results are graphed (*p < 0.05 vs. CON). (B & C) Western blot analysis for Smo (86 kDa), Gli3FL (full-length, 190 kDa) and Gli3R (repressor form, 83 kDa). Gapdh (36 kDa) expression was used as an internal control (n = 4/group). Data shown represent one of three experiments with similar results ((**B**) immunoblot/(**C**) band intensity). The mean ± SD results obtained by measuring the band density of three different blots are graphed (*p < 0.05, **p < 0.005 vs. CON). (**D**) Immunohistochemical staining for Gli3 in liver sections from the representative non-Tx and Tx rats at two and three weeks post transplantation (×40). The inserted image shows the representative liver section from the CON.

We assessed the expression of Hh-target genes in the Tx and non-Tx rats. Both the RNA and protein expression of *Smo*, a Hh receptor, decreased in the livers from the Tx rats at two weeks and was almost equivalent to the expression level of *Smo* in healthy liver at three weeks. In the non-Tx group, the expression of *Smo* was greatly increased at both the RNA and protein level (Fig. 2A–C). It was also examined whether the reduced expression of Smo resulted in the downregulation of the Gli family, the downstream signaling molecules of Smo. Compared with the non-Tx rats, the Tx rats had reduced mRNA expression of *gli3* (Fig. 2A). Western blot analysis revealed that CP-MSC-transplanted livers showed decreased expression of full-length Gli3 (Gli3FL, 190 kDa) and increased expression of the repressor form of Gli3 (Gli3R, 83 kDa) (Fig. 2B,C). The protein expression of Gli2 in the Tx rats was also significantly lower than in the non-Tx rats (Supplementary Fig. S2A and S2B). Immunostaining for Gli2 and Gli3 supported the more accumulation of Gli2- and Gli3-postive cells in the non-Tx rats than in the Tx rats (Fig. 2D, Supplementary Fig. S2C). The Gli2- or Gli3-positive cells were mainly found in the periportal hepatocytes and ductural cells in the livers of the non-Tx rats. There were fewer Gli2- or Gli3-positive cells in the livers of the Tx rats at two weeks, and hardly any positive cells were detected at three weeks. Interestingly, Gli3-positive cells were present in both hepatocytes and ductular-like cells, whereas Gli2-positive cells looked like ductural cells in the Tx rats at two weeks.

### Reduced fibrosis and expansion of hepatic progenitors in CP-MSC-transplanted livers

**Figure 3:**
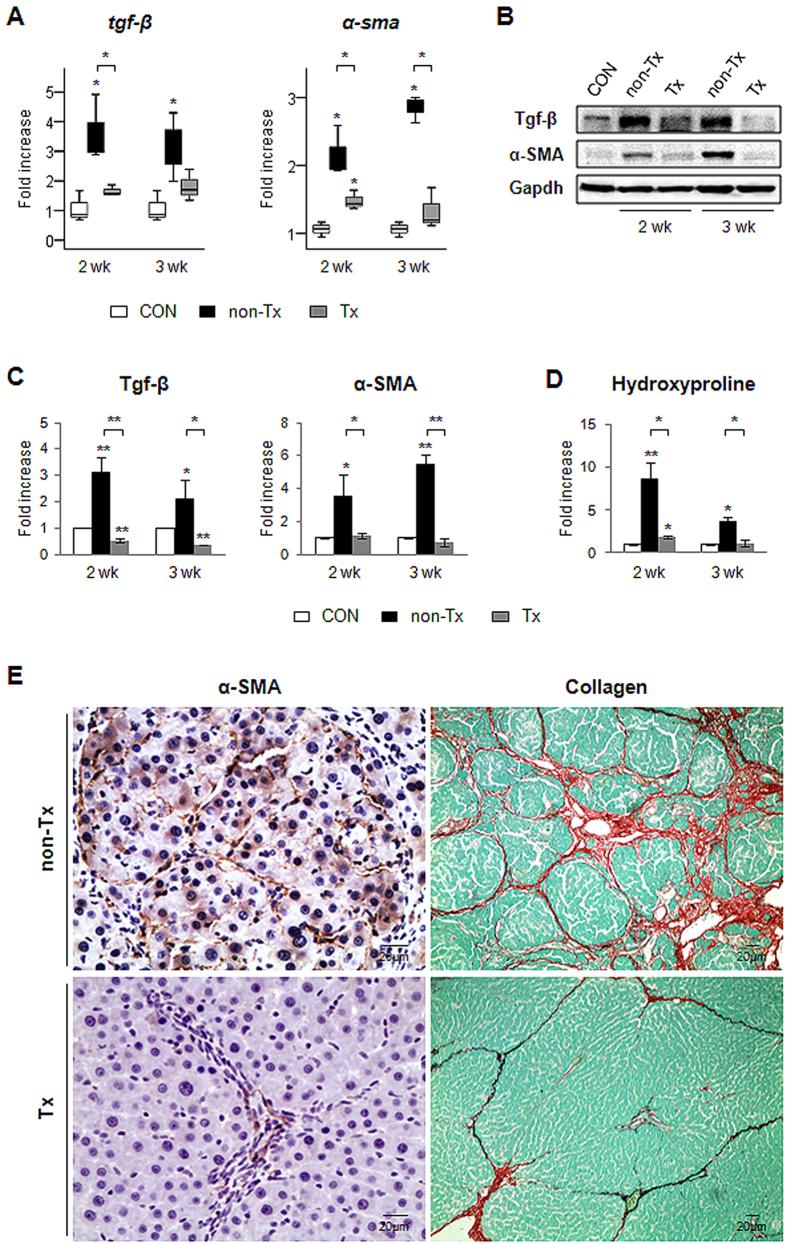
Reduced fibrosis in Tx rats. (**A**) QRT-PCR analysis for the profibrotic molecules including *tgf-β*, a fibrosis-stimulating factor, and *α-sma*, a fibrotic molecule, in the livers from the non*-*Tx, Tx and control (CON) group. Medians and ranges of results are graphed (*p < 0.05 vs. CON). (B & C) Western blot analysis for Tgf-β (25 kDa), α-SMA (42 kDa) and Gapdh (36 kDa, internal control) (n = 4/group). Data shown represent one of three experiments with similar results ((**B**) immunoblot/(**C**) band intensity). The mean ± SD results obtained by measuring the band density of three different blots are graphed (*p < 0.05, **p < 0.005 vs. CON). (**D**) Hepatic hydroxyproline content in all rats (n ≥ 4/grou*p*). The results are showed as the mean ± SEM (*p < 0.05, **p < 0.005 vs. CON). (**E**) Immunohistochemical staining for α-SMA (×40) and sirius red staining (×10) for examining collagen deposition were performed in liver sections from the representative non-Tx and Tx rat at two weeks.

Because Hh signaling is known to contribute to fibrosis through EMT^22^, it was determined whether changes in the expression of Hh between the Tx and non-Tx group were associated with fibrosis. Both RNA and the protein expression of *Tgf* (transforming growth factor)*-β*, a well-known EMT-stimulating factor^24^, and *α-SMA*, a marker of MF-HSCs^25^, were highly increased in livers treated with CCl_4_ without CP-MSC transplantation (non-Tx), compared to livers of control and Tx groups (Fig. 3A–C). The expression of both markers in the Tx rats was almost equivalent to that in the healthy livers. The activation of other EMT markers, such as *collagen 1α1*, *s100a4*, and *vimentin*, was downregulated in liver from the Tx rats compared to that from the non-Tx rats (Supplementary Fig. S3). Biochemical determination of hepatic hydroxyproline content, a quantitative measure of liver fibrosis, demonstrated that the Tx rats had significantly less liver fibrosis than the non-Tx rats (Fig. 3D). Immunostaining for α-SMA showed substantial accumulation of α-SMA-positive HSCs (brown-colored cells) in the non-Tx livers, whereas these cells were rarely detected in the Tx livers (Fig. 3E, Supplementary Fig. S4). Sirius red staining visualizing collagen deposition (red) confirmed that the Tx livers had less collagen fibrils in the pericellular and perisinusoidal spaces compared to the non-Tx livers (Fig. 3E, Supplementary Fig. S4). In addition, it was investigated whether the proliferation of liver progenitors decreased in CP-MSC-transplanted liver, because the severity in liver fibrosis paralleled the proliferation of progenitors^26^,^27^. Immunostaining for the progenitor markers, pancytokeratin (PanCK), a marker of activated cholangiocytes and liver progenitors^28^, and Sox9, a marker of new biliary cells during bile duct morphogenesis^29^, showed the significantly decreased accumulation of PanCK or Sox9-positve cells in livers of the Tx rats compared to the Non-Tx rats (Supplementary Fig. S5). These results suggested that the decreased activation of Hh signaling attenuated both of the fibrosis and the accumulation of progenitors in CP-MSC-transplanted livers.

### CP-MSC promotes inactivation of LX2 by suppressing Hh signaling

**Figure 4:**
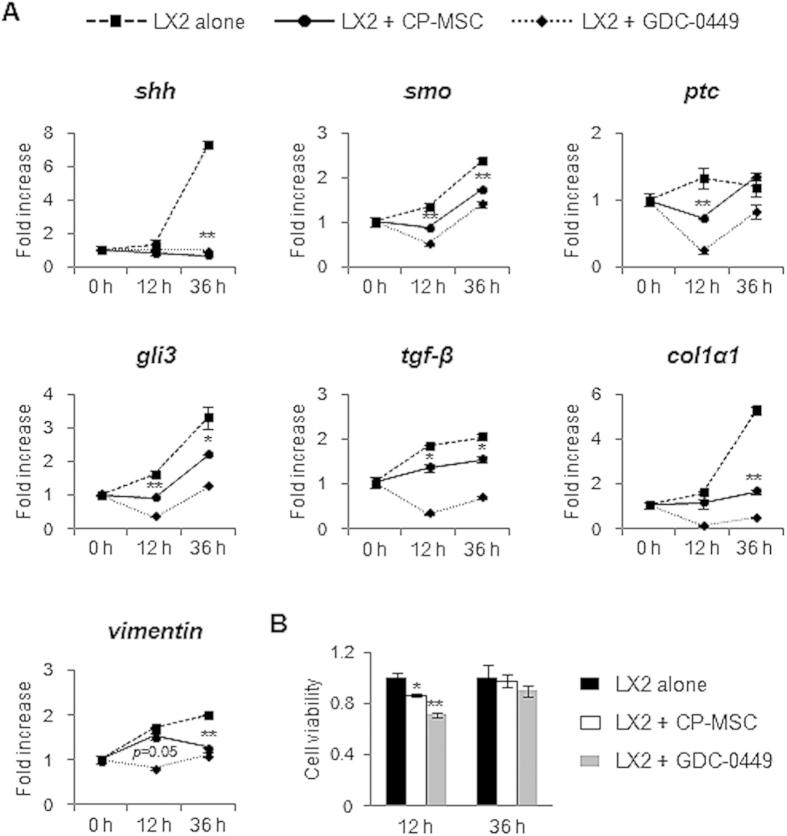
CP-MSCs suppress activation of hedgehog signaling and expression of profibrotic genes, and reduce cell viability of LX2. (**A**) QRT-PCR of the expression of Hh signals, including *shh*, *smo*, *ptc*, and *gli3*, and profibrotic genes, including *tgf-β, col1α1*, and *vimentin*, in LX2 mono-culture (alone, broken line), co-cultured LX2 with CP-MSCs (CP-MSC, solid line), and an LX2 mono-culture treated with 1 μM GDC-0449, an Smo antagonist (GDC-0449, dotted line), for 12 hours or 36 hours. The treatment time point was indicated as zero time. The level of gene expression in LX2 treated as above was shown as fold increase or decrease compared to that in fully activated LX2 at 48 hour (Supplementary Fig. S6). The mean ± SEM results obtained from three repetitive experiments are graphed (*p < 0.05, **p < 0.005 vs. mono-culture). (**B**) Cell number (growth) of mono-cultured LX2, co-cultured LX2 with CP-MSCs, and mono-cultured LX2 treated with 1 μM GDC-0449 for 12 or 36 hours was analyzed using the PrestoBlue™ assay. The mean ± SEM results obtained from three repetitive experiments are graphed (*p < 0.05, **p < 0.005 vs. mono-culture).

MF-HSC is a major cell type, which produce collagens and induce hepatic fibrosis in the injured liver^30^. Hh signaling is an essential factor regulating the activation and viability of MF-HSC^21^,^22^,^30^,^31^. Evidence for the lower expression of Hh signaling in CP-MSC-transplanted livers than fibrotic liver (Figs 1 and 2, Supplementary Fig. S1 and S2) suggested that CP-MSCs might influence the activation of MF-HSC by regulating Hh signaling. To assess the effect of CP-MSCs on MF-HSCs, fully activated LX2 (Supplementary Fig. S6), a human HSC line, was co-cultured with primary CP-MSCs for 12 and 36 hours in a Transwell inserts system. The expression of Hh signaling and fibrotic genes was compared to that of mono-cultures of LX2. GDC-0449, an *smo* inhibitor, was previously shown to suppress the activation of MF-HSCs and fibrosis by inhibiting Hh signaling^32^. GDC-0449 (1 μM) was employed as a positive control for Hh inhibition. LX2 in mono-culture showed robust increase of expression of Hh signaling and profibrotic genes (Fig. 4A). GDC-0449 effectively reduced the expression of both Hh signaling and profibrotic genes. Co-culture influenced the expression of both Hh signaling and profibrotic genes in the LX2, like GDC-0449. The level of *shh* showed baseline expression during co-culture. The mRNA expression of Hh-target genes, *smo*, *ptc*, and *gli3* in the LX2 was downregulated during co-culture compared to mono-culture. The expression of profibrotic genes, such as *tgf-β*, *col1α1*, and *vimentin*, in the co-cultured LX2 was lower than that in the mono-culture. The inhibitory actions of the CP-MSCs and GDC-0449 on the LX2 were diminished at 36 hour. In addition, both CP-MSCs and GDC-0449 reduced the number of LX2, decreasing cell counts <15% (0.87 ± 0.009) and <30% (0.71 ± 0.015), respectively, at 12 hours and this effect of CP-MSCs and GDC-0449 on LX2 viability was regressed at 36 hours (Fig. 4B).

### CP-MSCs express miRNA-125b

Previous studies have shown that MSCs harbor miRNAs, which have gene regulating effects^13^,^14^, and our own results showed the inhibitory effect of CP-MSCs on the expression of Hh signals *in vitro* and *in vivo*, the present study therefore investigated whether CP-MSCs retained miRNAs targeting Hh signaling molecules.

**Figure 5:**
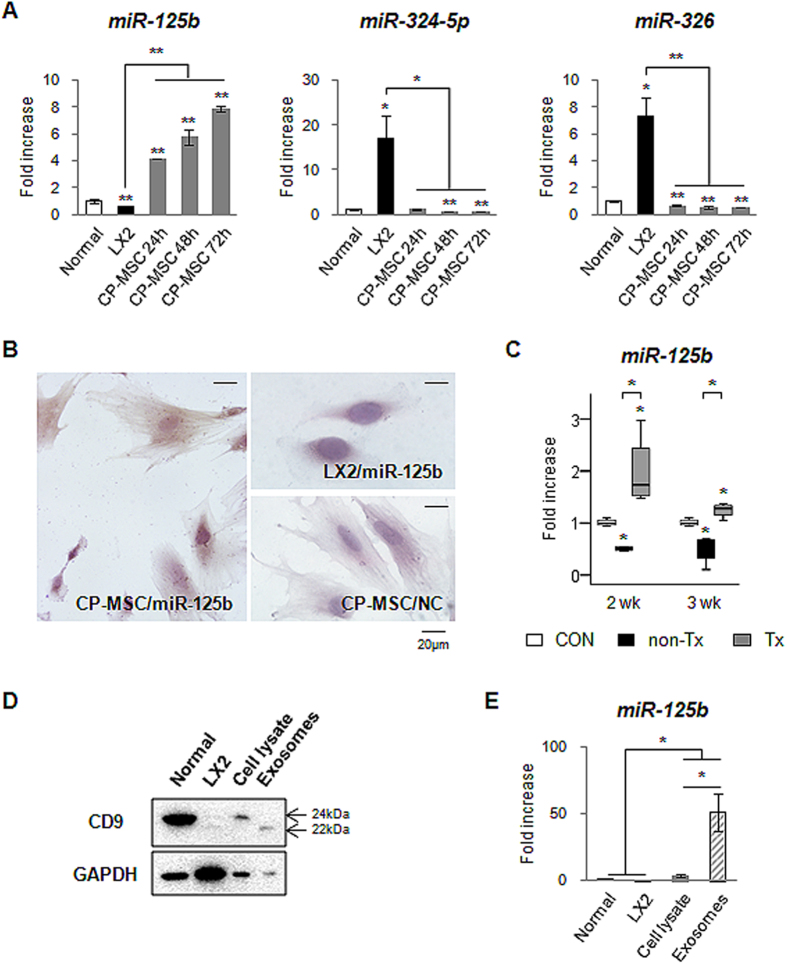
MiRNA-125b is expressed in CP-MSCs. (**A**) QRT-PCR analysis of the expression of miRNA-125b, miRNA-324-5*p* and miRNA-326 in human normal liver (Normal), LX2 (human HSC line) and human CP-MSCs cultured during 72 hours. The mean ± SEM results obtained from three repetitive experiments are graphed (*p < 0.05, **p < 0.005 vs. Normal). (**B**) *In situ* hybridization (ISH) with miRNA-125b probe (50 nM) in CP-MSCs and LX2 cultured for 24 hours. ISH with scrambled-miRNA probe (50 nM) in CP-MSCs as a negative control (NC). (**C**) QRT-PCR analysis of hepatic expressions of miRNA-125b in the livers from all rats. Medians and ranges of results are graphed (*p < 0.05 vs. CON). (**D**) Representative western blot analysis for CD9 (22 or 24 kDa), an exosomal marker, and GAPDH (36 kDa) in lysates of normal human liver (normal), LX2, and CP-MSCs (Cell lysate) and exosomes from CP-MSC-CM (Exosomes). (**E**) QRT-PCR analysis for miRNA-125b expression in Normal liver, LX2, CP-MSC lysates, and exosomes produced by CP-MSCs. The mean ± SEM results obtained by three repetitive experiments are graphed (*p < 0.05).

It was previously reported that miRNA-125b, miRNA-324-5p and miRNA-326 targeted Hh target genes in human medulloblastoma cell lines^33^. Hence, we examined which types of miRNAs were expressed in CP-MSCs. CP-MSCs showed highly increased expression of miRNA-125b targeting s*mo* compared to human healthy liver and activated LX2 (*p* < 0.005). The expression of both miRNA-324-5p and miRNA-326 inhibiting *gli1* was lower in CP-MSCs than in healthy human liver or LX2 (Fig. 5A). The expression of miRNA-125b showed a gradual increase, peaking at 72 hours followed by an eventual decline during CP-MSCs culture (Supplementary Fig. S7A). ISH analysis showed the expression of miRNA-125b (brown-colored dots) in cytosol of CP-MSCs (Fig. 5B). That expression was not detected in either LX2 or CP-MSCs hybridized with scrambled-miRNA probe (negative control: NC) (Fig. 5B). In addition, Tx livers showed upregulation of miRNA-125b (1.98 ± 0.34-fold increase compared to healthy liver), whereas non-Tx livers showed downregulation of miRNA-125b (0.50 ± 0.02-fold decrease compared to healthy liver) at two weeks post-transplantation (Fig. 5C). The differential expression of miRNA-125b in the non-Tx and Tx livers was sustained until three weeks post transplantation. To examine how CP-MSCs or CP-MSC-transplanted liver expressed higher amount of miRNA-125bs, we isolated exosomes from culture medium (CM) of CP-MSCs cultured for 48 hours in exosome-depleted medium. The isolated exosomal fractions from CP-MSC lysate or CP-MSC-CM were confirmed by the expression of CD9 which is an exosomal membrane protein with processed and unprocessed forms (Fig. 5D). QRT-PCR analysis revealed that miRNA-125b expression was greater in the exosomes from CP-MSC-CM than in human normal liver and LX2 (51.09 ± 13.97-fold increase compared to healthy live; 69.47 ± 18.99-fold increase compared to LX2). The lysate of CP-MSCs also showed increased level of miRNA-125b compared to human normal liver and LX2 (Fig. 5E). These results suggest that the exosomes in CP-MSCs contained miRNA-125b, which inhibited the expression of Hh-target genes.

### MiRNA-125b by CP-MSCs suppresses activation of HSCs by abrogating Hh expression

**Figure 6:**
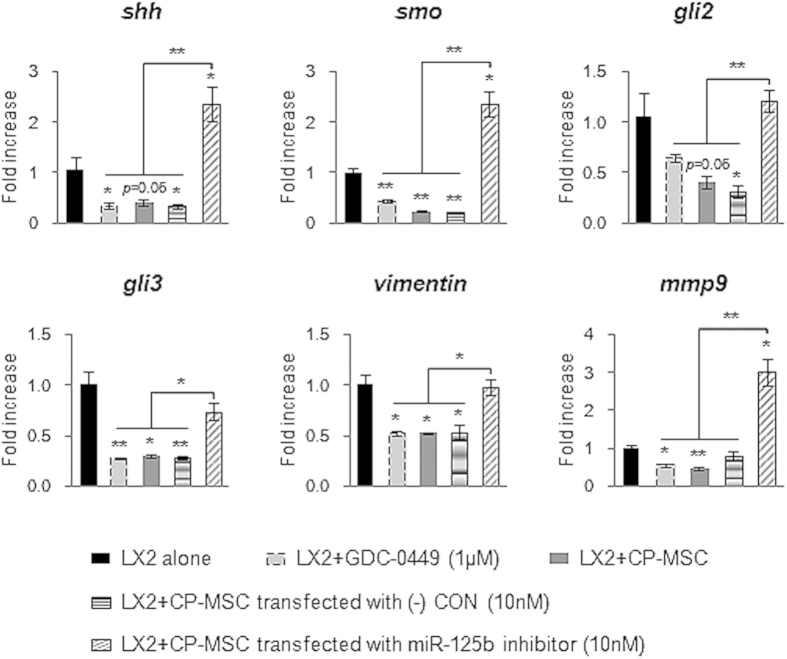
MiRNA-125b-downregulated CP-MSCs fail to downregulate expression of Hh signaling and profibrotic genes in LX2. QRT-PCR analysis of the expression of Hh signals, including *shh*, *smo*, *gli2*, and *gli3*, and profibrotic genes, including *vimentin* and *mmp9*, in LX2 mono-culture (alone), LX2 treated with 1 μM GDC-0449 (Smo antagonist), and co-cultured LX2 with CP-MSCs (CP-MSC), CP-MSC having miRNA-125b inhibitor (CP-MSC miRNA-125b inhibitor), or CP-MSC having scrambled-miRNA inhibitor (CP-MSC (-) CON) for 12 hours. The mean ± SEM results obtained from three repetitive experiments are graphed (*p < 0.05, **p < 0.005 vs. LX2 alone).

To confirm whether the inhibitory effect of CP-MSCs on Hh signaling was caused by miRNA-125b, LX2 was co-cultured with CP-MSCs in which miRNA-125b expression was inhibited. CP-MSCs were transfected with 10 nM of human miRNA-125b inhibitor or scrambled-miRNA inhibitor not disturbing any miRNA expression, demonstrating transfection specificity. The miRNA-125b inhibitor effectively reduced the expression of miRNA-125b in CP-MSCs at 12 to 24 hours after transfection, whereas the false inhibitor affected neither the expression of miRNA-125b nor the cell viability of CP-MSCs (Supplementary Fig. S7B). The expression level of miRNA-125b in CP-MSCs transfected with the negative control was equivalent to that in CP-MSCs without transfection. After miRNA-125b was successfully downregulated in CP-MSCs at 12 hours, we transferred the Transwell inserts containing CP-MSCs onto wells with fully activated LX2, and then co-cultured those cells for 12 hours. Subsequently, the expression of the Hh signaling molecules, *shh*, s*mo*, *gli2,* and *gli3*, and the profibrotic genes, *vimentin* and *mmp9,* was still higher in co-cultured LX2 with miRNA-125b-downregulated CP-MSCs than in any other treatment group, such as LX2 cultured with GDC-0449, CP-MSCs, or CP-MSCs having a negative control inhibitor (Fig. 6).

**Figure 7:**
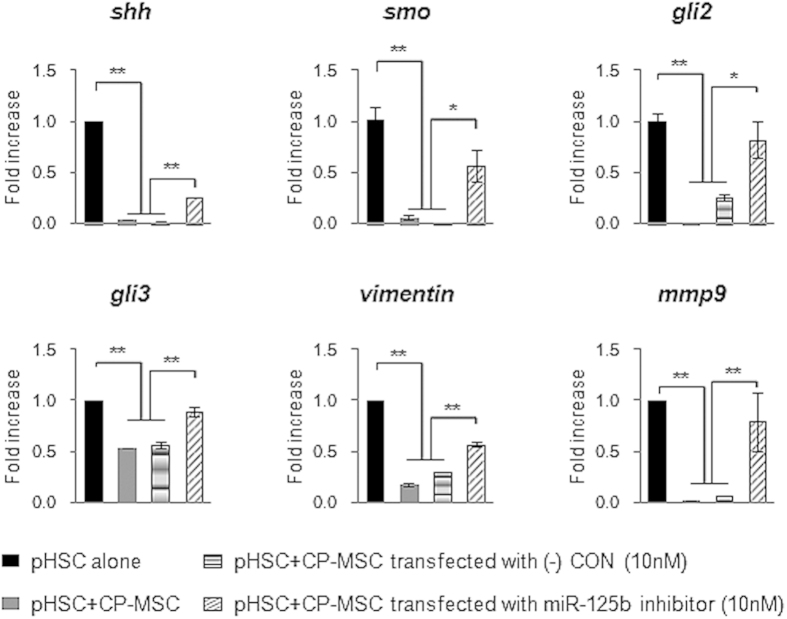
Hh signaling and profibrotic genes were highly expressed in activated primary HSCs co-cultured with CP-MSCs containing miRNA-125b inhibitor. QRT-PCR analysis of the expression of Hh signals, including *shh*, *smo*, *gli2*, and *gli3*, and profibrotic genes, including *vimentin* and *mmp9*, in mono-cultured primary HSCs isolated from rats with CCl_4_-induced fibrosis (alone), and co-cultured primary rat HSCs with CP-MSCs (CP-MSC), CP-MSC having miRNA-125b inhibitor (CP-MSC miRNA-125b inhibitor), or CP-MSC having scrambled-miRNA inhibitor (CP-MSC (-) CON) for 12 hours. The mean ± SEM results obtained from three repetitive experiments are graphed (*p < 0.05, **p < 0.005 vs. pHSC alone).

To show whether the inhibitory action of miRNA-125b in the activation of HSCs was relevant to physiological condition, we isolated primary HSCs from chronically damaged liver of rats (Supplementary Fig. S8). These activated HSCs were co-cultured with CP-MSCs transfected with scrambled or miRNA-125b inhibitor. The expression of the Hh signaling molecules, *shh*, s*mo*, *gli2,* and *gli3*, and the profibrotic genes, *vimentin* and *mmp9,* was downregulated in primary HSCs co-cultured with CP-MSCs or scrambled inhibitor-transfected CP-MSCs, whereas the expression of those genes was upregulated in primary HSCs co-cultured with miRNA-125b-inhibited CP-MSCs (Fig. 7).

**Figure 8:**
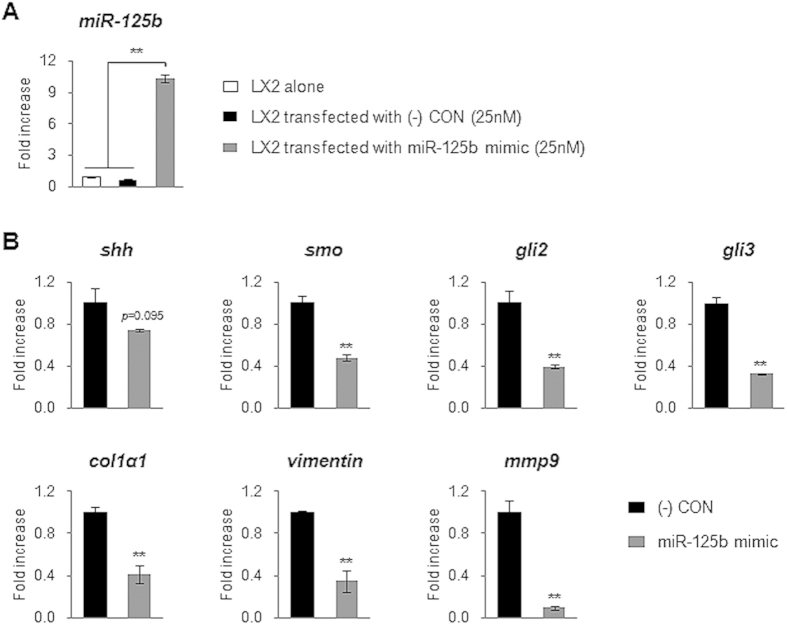
MiRNA-125b regulates expression of Hh signaling and profibrotic genes in LX2. (**A**) QRT-PCR analysis of miRNA-125b expression in LX2 or transfected LX2 with miRNA-125b mimic (25 nM) or scrambled-miRNA mimic ((-) CON, 25 nM) as a negative control for 24 hours. The mean ± SEM results obtained from three repetitive experiments are graphed (**p < 0.005). (**B**) QRT-PCR analysis for genes of Hh signaling, including *shh*, *smo*, *gli2* and *gli3*, and profibrotic genes, including *col1α1*, *vimentin* and *mmp9*, in LX2 transfected with either scrambled-miRNA mimic ((-) CON) or miRNA-125b mimic for 24 hours. The mean ± SEM results obtained from three repetitive experiments are graphed (**p < 0.05 vs. (-) CON).

In addition, we transfected directly miRNA-125b mimic into LX2 in order to assess the specific function of miRNA-125b on LX2 activation. LX2 transfected with miRNA-125b mimic showed increased expression of miRNA-125b after transfection (Fig. 8A), followed by decreased expression of Hh pathway genes, *smo*, *gli2*, and *gli3,* and profibrotic genes, *col1α1*, *vimentin*, and *mmp9* (Fig. 8B).

Therefore, these findings suggest that miRNA-125b produced by CP-MSCs inhibits the activation of MF-HSCs by regulating Hh expression.

## Discussion

The Hh pathway is a well-known factor regulating liver reconstitution^17^,^18^,^23^. Our findings demonstrate for the first time that CP-MSCs harbor miRNA-125b inhibiting expression of *Smo,* and attenuate Hh activation in CP-MSC-transplanted liver, suggesting that Hh signaling plays a pivotal role in the regenerative effects of CP-MSCs on liver with chronic damage. Hh signaling regulated by CP-MSCs led to reduced fibrosis and liver restoration. Interestingly, the effect of the CP-MSCs on the activation of MF-HSCs is similar to that of GDC-0449, a *Smo* inhibitor, *in vitro*. *Smo* is regarded as an effective therapeutic target to control Hh signaling in various diseases, including cancer and liver disease^32^,^34^,^35^. However, *Smo* antagonists, such as GDC-0449 and cyclopamine, are difficult to apply in a clinical setting due to limitations, such as induction of resistance, pH-dependent efficiency, and side effects on normal cells^36^,^37^. Our results show that CP-MSCs effectively controlls Hh signaling by inhibiting and regulating Hh signaling, thereby contributing to liver regeneration.

MiRNA-125b is complementary to the 3′-untranslated region on the *smo* gene conserved in human and mouse^33^. One study reported that miRNA-125b is rich in MSCs derived from human BM, as well as in human liver resident stem cells (HLSCs)^15^. Moreover, MSCs, including BM-MSCs and HLSCs, were shown to release exosomes or MVs containing specific miRNAs^15^,^16^. In line with previous findings, we present evidence that miRNA-125b is highly expressed in CP-MSCs, but not in LX2 (Fig. 5A,B), and that it is retained in the exosomes released from CP-MSCs (Fig. 5E). The expression of miRNA-125b was also significantly increased in the CP-MSC-transplanted livers, and upregulation of miRNA-125b led to a reduction of *Smo*. However, miRNA-125b was greatly reduced with an increase of *Smo* in the fibrotic liver compared to healthy and CP-MSC-transplanted livers. In addition, our co-culture experiments demonstrated that CP-MSCs inhibited the expression of Hh signaling and profibrotic genes in LX2 and primary HSCs, whereas miRNA-125b-suppressed CP-MSCs did not induce such changes in those cells (Figs 4, 6 and 7). Both CP-MSCs and GDC-0449 also reduced the viability of LX2 at 12 hours post-treatment at which time they effectively suppressed the expression of Hh signaling and profibrotic genes in LX2 (Fig. 4B). Because Hh signaling is known as a viable factor for myofibroblastic HSCs^21^,^31^, it confirms our findings that CP-MSCs lead to miR-125b-suppressed Hh signaling, which contributes to the decreased viability of myofibroblasts, activated HSCs. In addition, several studies reported that human BM-MSCs inhibited the proliferation, but induced the apoptosis of human activated HSCs and primary rat HSCs in co-culture system, simultaneously reducing the expression of fibrotic genes^38^,^39^. In line with their finding, our data suggest the possibility that CP-MSCs might inhibit proliferation or induce apoptosis of LX2. Taken together, those results support our hypothesis that CP-MSCs release exosomes or MVs containing miRNA-125b into target cells, such as Hh-responsive HSCs, and hinder the activation of Hh signaling by inhibiting *Smo* expression, eventually alleviating hepatic fibrosis.

Although the protein level of Smo in the CP-MSC-transplanted livers was almost equivalent to the expression level of Smo in controls, the mRNA level showed a 2.74 ± 0.10-fold increase compared to the controls at two weeks (*p* < 0.05) (Fig. 2A–C). It is possible that animal miRNAs induce gene silencing by translational inhibition, followed by degradation of their target mRNAs by deadenylation^40^. Downregulation of the Smo protein influenced the expression and the translocation of the downstream transcriptional factors, Glis, which are the final destination of the Hh signaling pathway. The function of individual Glis in the liver remains elusive, and there is substantial interest in determining which types of Glis are related to fibrogenesis. The expression of both *Gli2* and *Gli3* notably increased during hepatic fibrogenesis, whereas their expression was reduced to baseline expression levels after CP-MSC-transplantation. Gli2 and Gli3 have activator and repressor domains in the C- and N-terminal region, respectively, whereas Gli1 has only an activator domain^41^. C-terminal-processed Gli3 acts as a repressor (Gli3R), which is abundant in CP-MSC-transplanted livers. In addition, immunostaining for Gli2 and Gli3 showed the robust accumulation of Gli2- or Gli3-positive periportal hepatocytic or ductular cells in the non-Tx group, compared to the Tx group. Previous studies suggested that Gli2-positive hepatocytes might be immediate progeny of Gli2-positive progenitors^19^,^23^. Hence, it is possible that Gli2- or Gli3-positive cells might be derived from Gli2 or Gli3-positive progenitors, which were evident in the non-Tx rats. In addition, both miRNA-324-5p targeting *gli1* and miRNA-326 regulating the expression of *Smo* and *Gli1* by cooperating with miRNA-324-5p^33^ were highly expressed in LX2, but not in CP-MSC (Fig. 5A). These data are consistent with previous findings that *Gli1* is sparse in HSCs and not increased upon activation of HSCs^42^.

The current study found intriguing evidence that the regulation of the Hh ligands, Shh and Ihh, differs during liver regeneration. Under chronic and severe liver injury, hepatocytes undergo massive apoptosis, release Shh, and stimulate Hh-responsive cells, such as immature ductular cells or HSCs^19^,^20^. These eventually proliferate and undergo transformation to a myofibroblastic phenotype^19^,^20^. These Hh-responsive cells also produce Shh, activate Hh signaling pathways in an autocrine manner, and promote a Hh-enriched hepatic microenvironment, eventually exacerbating hepatic fibrosis^18^,^21^. The increased expression of Shh in cirrhotic liver gradually decreased following a reduction in Hh-responsive cells in the CP-MSC-transplanted livers. It is possible that abundant miRNA-125b in the CP-MSCs and in the CP-MSC-transplanted liver downregulates the Hh activator Smo and Glis. The resulting reduction of Hh activators may decrease the population of Hh-responsive cells and result in the inactivation of these cells, eventually leading to a fall in Shh production and a reduction in fibrosis. In the present study, the expression of another Hh ligand, Ihh, was greatly increased in the CP-MSC-transplanted livers. The expression of Ihh was also apparent in progenitor (oval shape) and HSC-like cells, and Shh was expressed in apoptotic hepatocytes in the fibrotic tract. This differential expression of Ihh and Shh indicate that they may exert a distinct effect on the process of liver repair. The slight increase of *Smo* and *Gli*s in the Tx rats compared with the control rats also seemed to be caused not only by Shh but also by Ihh. In line with our findings, Omenetti *et al.* reported that *Ihh* expression declined at one week after bile duct ligation, causing biliary fibrosis, but gradually increased during reversal of biliary fibrosis^17^. On the other hand, *Shh* expression was elevated during fibrosis, and steadily dropped during recovery. Taken together, Ihh seems to be involved in liver reconstitution by mediating the mesenchymal-to-epithelial transition, a reversal process of EMT. However, further studies are required to demonstrate the specific function of Shh and Ihh related to Hh-target genes, Glis.

Our results demonstrated that miRNA-125b produced by CP-MSCs regulated the expression of Hh signaling which promoted the regression of fibrosis, eventually contributing to liver regeneration. These findings point to the underlying mechanism for the therapeutic effects of CP-MSCs in chronic liver diseases, and may help the development of novel diagnostic and therapeutic approaches to prevent and treat liver fibrosis.

## Methods

### Preparation of human chorionic plate-derived mesenchymal stem cells (CP-MSCs)

Normal term (gestation ≥37 weeks) placentas were donated by women who provided written informed consent for research purpose. Collection and utilization of samples were approved by the Institutional Review Board of CHA General Hospital, Seoul, Korea. Human CP-MSCs were isolated from chorionic plate of placentas as previously reported^6^. The human experiments were performed in accordance with the approved guidelines.

### Experimental animals

Murine models of liver disease were constructed as previously described^6^. Briefly, six-week-old male Sprague-Dawley rats received 1.6 g/kg body weight of CCl_4_ (Sigma-Aldrich) dissolved in corn-oil, intraperitoneally. To induce liver fibrosis, CCl_4_ was injected twice weekly for 9 weeks. At Cellular membranes of human CP-MSCs were labeled with PKH26 Red Fluorescent Cell Linker (Sigma-Aldrich) and the PKH26-labeled CP-MSCs (2 × 10^6^ cells) were transplanted directly into the right lobe of CCl_4_-injured livers at a depth of 5 mm. The PKH26-labeled CP-MSCs were successfully engrafted, which was confirmed by fluorescence microscopy and immunohistochemical staining for human-specific nuclei^6^. CP-MSC-transplanted and non-transplanted CCl_4_-injured rats were sacrificed 2 and 3 weeks after transplantation (n ≥ 4/group). As controls, rats received the same volume of corn-oil intraperitoneally (n = 4). Animal care and surgical procedures were approved by the Pusan National University Institutional Animal Care and Use Committee and carried out in accordance with the provisions of the National Institutes of Health (NIH) Guide for the Care and Use of Laboratory Animals.

### Cell experiment

Primary CP-MSCs and human HSC line LX2 (provided by Dr. Jeong, KAIST, KOREA) were cultured in α-MEM (Gibco) supplemented with 10% FBS (Gibco), GlutaMAX™ (Gibco) and penicillin/streptomycin (Gibco) at 37 °C in a humidified atmosphere containing 5% CO_2_. To biochemical analysis of gene expressional changes, LX2 at 70–80% confluence were starved in medium containing no FBS for 6 hours. Activation of LX2 was verified by examining the expression of profibrotic and Hh signaling genes at 24, 48, and 72 hour post addition of FBS. These experiments were repeated three times. Based on the data of gene expression, LX2 was considered to be fully activated at 48 hours (Supplementary Fig. S6). Fully activated LX2 was cultured alone, co-cultured with CP-MSCs, or treated Vismodegib (1 μM GDC-0449; Selleck Chemicals), Smo antagonist. Co-cultures between LX2 and CP-MSCs were conducted using Transwell inserts (Corning Inc., NY) in which culture medium was diffusible but cells were not permeable. CP-MSCs (2 × 10^6^ cells/insert) cultured for 24 hours were used in the co-culture experiments. All co-culture experiments were performed with LX2 in the bottom wells, and CP-MSCs in the top wells using CP-MSC-conditioned medium (CM). Insert chamber with CP-MSCs were transferred into co-culture system and cultured for 12 or 36 hours. The control human liver tissues were generously provided from Dr. Anna Mae Diehl and Steve S. Choi (Duke University Medical Center) and those controls were obtained from residual healthy liver tissues of five donor livers that were utilized for split liver transplantation at Duke University Hospital in accordance with NIH and Duke institutional guidelines for human subject research. To evaluate the effect of CP-MSCs and GDC-0449 on cell viability and proliferation of LX2, we performed PrestoBlue™ Cell Viability assay according to the manufacturer’s instructions in 96-well plate. PrestoBlue™ reagent (Invitrogen) was added directly to the live cells in culture medium, and then those cells were incubated for 30 minutes at 37 °C. Absorbance was measured at 560 nm with correction at 600 nm using GloMax Microplate reader (Promega). The cell viability was expressed as ΔOD (OD_560_-OD_600_). Cell experiments were repeated three times and data were shown as the mean ± standard error of mean (SEM).

### *In situ* hybridization (ISH) for miRNA-125b in cells

1 × 10^5^ cells/well (6-well plate) of CP-MSCs and LX2 were seeded and cultured in growth medium for 24 hours. To perform ISH, cells were fixed, prehybridized in microRNA ISH buffer (Exiqon, Vedbaek, Denmark) at 55 °C for 2 hours, and hybridized with 5′-digoxigenin (DIG)-labeled LNA™ microRNA probe (Exiqon, Vedbaek, Denmark) detecting hsa-miRNA-125b-5p or scramble-miRNA at 55 °C overnight. Anti-DIG-peroxidase antibody (Roche) and TSA plus DIG amplification were employed in the detection procedure, according to the manufacturer’s instructions (PerkinElmer, inc., Waltham, MA, US). DAB was used as a substrate for peroxidase to visualize staining as brown color.

### Isolation and analysis of exosomal miRNA-125b of CP-MSCs

Exosomes were isolated from CP-MSCs and CP-MSC-CM by differential centrifugation as previously describe^43^,^44^. Briefly, CP-MSCs were cultured in α-MEM deprived of FBS for 48 hours to stimulate the exosome release. The culture medium was collected and centrifuged at 300 g for 10 minutes, followed by a centrifugation at 2,000 g for 20 minutes to remove the cells. The cell-free supernatants were then centrifuged at 10,000 g for 30 minutes using ultracentrifuge (Optima L-90K, Beckman Coulter), to remove the cell debris. After this step, the supernatants were ultracentrifuged at 100,000 g for 70 minutes twice and the pellet was washed with phosphate buffered saline (PBS). All steps were conducted at 4 °C. Exosome-containing RNA and protein was purified from the pellet using TRIzol reagent according to the manufacturer’s instructions. Western blot analysis for CD9 (diluted 1:1000; Abcam) was performed to prove the exosome existence.

### Transfection of miRNA-125b inhibitor into CP-MSCs

CP-MSCs (1 × 10^5^ cells/well) cultured for 24 hours were transfected with 10 nM of miRNA-125b inhibitor (AccuTarget™ human miRNA-125b inhibitor, Bioneer corp., Korea) or 10 nM of miRNA inhibitor negative control (miRNA inhibitor negative control #1, Bioneer corp., Korea), according to the manufacturer’s instructions. Before treating cells with miRNA inhibitors, the diluted miRNA-125b inhibitor or negative control inhibitor in Opti-MEM® I reduced serum medium (Gibco) was incubated with the diluted Lipofectamine RNAiMAX transfection reagent (Invitrogen) in Opti-MEM® I for 20 minutes at RT to make the transfection complexes. After adding the transfection complexes to the cells, cells were incubated at 37 °C in a humidified atmosphere containing 5% CO_2_ for 12 or 24 hours. Cell viability at 12 and 24 hour post transfection was determined to be >95%, as established by trypan blue exclusion.

The transfected CP-MSCs with miRNA-125b or negative control were transferred into co-culture system and cultured with fully activated with LX2 for 12 hours. Although the expression level of miRNA-125b in CP-MSCs were significantly downregulated at 12 and 24 hours after transfection (Supplementary Fig. S7B), the transfected cells for 12 hours were used in the co-culture experiments, considering the suppressive effect of the inhibitor in the expression of miRNA-125b. Cell experiments were repeated three times and data were shown as the mean ± SEM.

### Transfection of miRNA-125b mimic into LX2

LX2 (1.5 × 10^5^ cells/well) cultured for 24 hours were transfected with 10 nM of miRNA-125b mimic (AccuTarget™ human miRNA-125b mimic, Bioneer corp., Korea) or 10 nM of miRNA mimic negative control (miRNA mimic negative control #1, Bioneer corp., Korea) for 24 hours, according to the manufacturer’s instructions and briefly described above.

### Primary HSC isolation from rat with CCl_4_-induced liver fibrosis

Five-week-old male Sprague-Dawley rats received CCl_4_ mixed with corn oil (CCl_4_: corn oil = 1:1) intraperitoneally twice weekly for 5 weeks. 0.2 ml/100 g of CCl_4_ was injected twice for 2 weeks, and then 0.1 ml/100 g of CCl_4_ was injected twice for 3 weeks^45^. At 3 days after the last injection, rats were sacrificed. Liver morphology and fibrosis were confirmed by H&E staining, immunostaining for α-SMA, and sirius red staining (Supplementary Fig. S8). Primary HSCs were isolated from the fibrotic livers as previously described^46^,^47^. Briefly, rats were anesthetized with zoletil 50 (5 mg/kg body weight, Virbac S.A., France) to immobilize in the recumbent position on a treatment table, and inferior vena cava was cannulated under aseptic conditions. Livers were perfused *in situ* with EGTA and collagenase (Roche) to disperse the cells. Primary HSCs were isolated by differential centrifugation on OptiPrep (Sigma-Aldrich) density gradient, and located on the upper layer of 11.5% OptiPrep at a purity of over 98%. Isolated HSCs were cultured in Minimum Essential Medium alpha (MEM α, Gibco) supplemented with 10% fetal bovine serum (FBS, Gibco), GlutaMAX™ (Gibco) and penicillin/streptomycin (Gibco) at 37 °C in a humidified atmosphere containing 5% CO_2_. The cell viability was determined to >95% by trypan blue staining.

### Statistical analysis

QRT-PCR data from tissue are expressed as median and range to show distributions of individuals. Comparisons between groups were assessed using Mann-Whitney U-test performed with IBM SPSS Statistics 21 software (Release version 21.0.0.0, IBM Corp., Armonk, NY) and differences were considered significant when *p* < 0.05.

Results of protein analysis in tissue and RNA analysis in cells are shown as the mean ± SD and mean ± SEM, respectively, obtained from three different experiments. Statistical significances between control and treated groups or between treated subgroups were analyzed by the unpaired two-sample Student’s t-test and *p* values < 0.05 were considered as significant.

### RNA analysis, Western blot analysis, and Hydroxyproline assay

See Supplementary Information online.

## Additional Information

**How to cite this article**: Hyun, J. *et al.* MicroRNA125b-mediated Hedgehog signaling influences liver regeneration by chorionic plate-derived mesenchymal stem cells. *Sci. Rep.* **5**, 14135; doi: 10.1038/srep14135 (2015).

## Supplementary Material

Supplementary Information
