# Supplementary material for: MicroRNA125b-mediated Hedgehog signaling influences liver regeneration by chorionic plate-derived mesenchymal stem cells
Source: Sci Rep. 2015 Sep 15;5:14135. doi: 10.1038/srep14135 (PMC4569897; doi:10.1038/srep14135)
Supplement: Supplementary Information [file srep14135-s1.pdf]

MicroRNA125b-mediated Hedgehog signaling influences liver regeneration by chorionic plate-derived mesenchymal stem cells

Jeongeun Hyun[1], Sihyung Wang[1], Jieun Kim[1], Gi Jin Kim[3], and Youngmi Jung[1,2,*]

[1]Department of Integrated Biological Science, [2]Department of Biological Science, College of Natural Science, Pusan National University, Pusan, 609-735, Korea

[3]Department of Biomedical Science, CHA University, Seoul, 135-081, Korea

[*]Tel.: +82-51-510-2262; Fax: +82-51-581-2962; E-mail: y.jung@pusan.ac.kr

Supplementary Information

Supplementary Figure S1. Expression of Shh and Ihh in non-Tx and Tx rats at 3 weeks.

Supplementary Figure S2. Reduced expression of Gli2 in Tx rats.

Supplementary Figure S3. Decreased expressions of EMT-related genes during liver regeneration.

Supplementary Figure S4. Comparison of fibrosis in the Tx rats and Non-Tx rats at 3 weeks post transplantation.

Supplementary Figure S5. Decreased accumulation of hepatic progenitors in liver from Tx rats.

Supplementary Figure S6. Increased expression of Hh signaling and profibrotic genes in LX2 cell during culture.

Supplementary Figure S7. The expressional changes of miRNA-125b in CP-MSCs with or without miRNA-125b inhibitor.

Supplementary Figure S8. Liver is chronically damaged by CCl_4 in rats.

Supplementary Table S1. Quantitative Real-Time PCR Primer Sequences of rat.

Supplementary Table S2. Quantitative Real-Time PCR Primer Sequences of human.

Supplementary Methods including RNA analysis, Western blot analysis, and Hydroxyproline assay

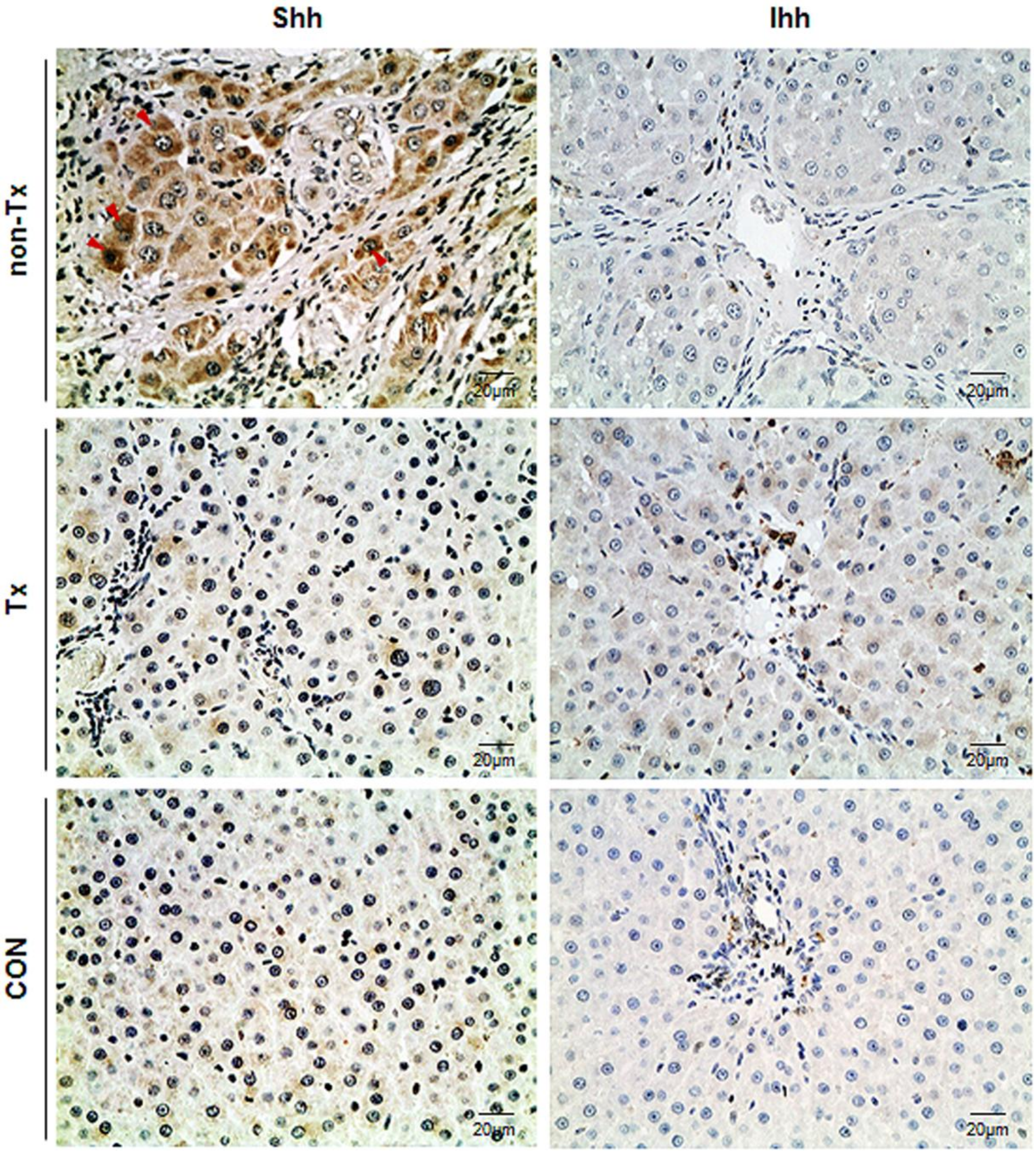

Supplementary Figure S1. Expression of Shh and Ihh in non-Tx and Tx rats at 3 weeks. Immunohistochemistry for Shh and Ihh in liver sections from the representative non-Tx and Tx rats at 3 weeks after transplantation, and control (CON) rats (×40). Shh-positive hepatocytes are indicated by arrowhead.

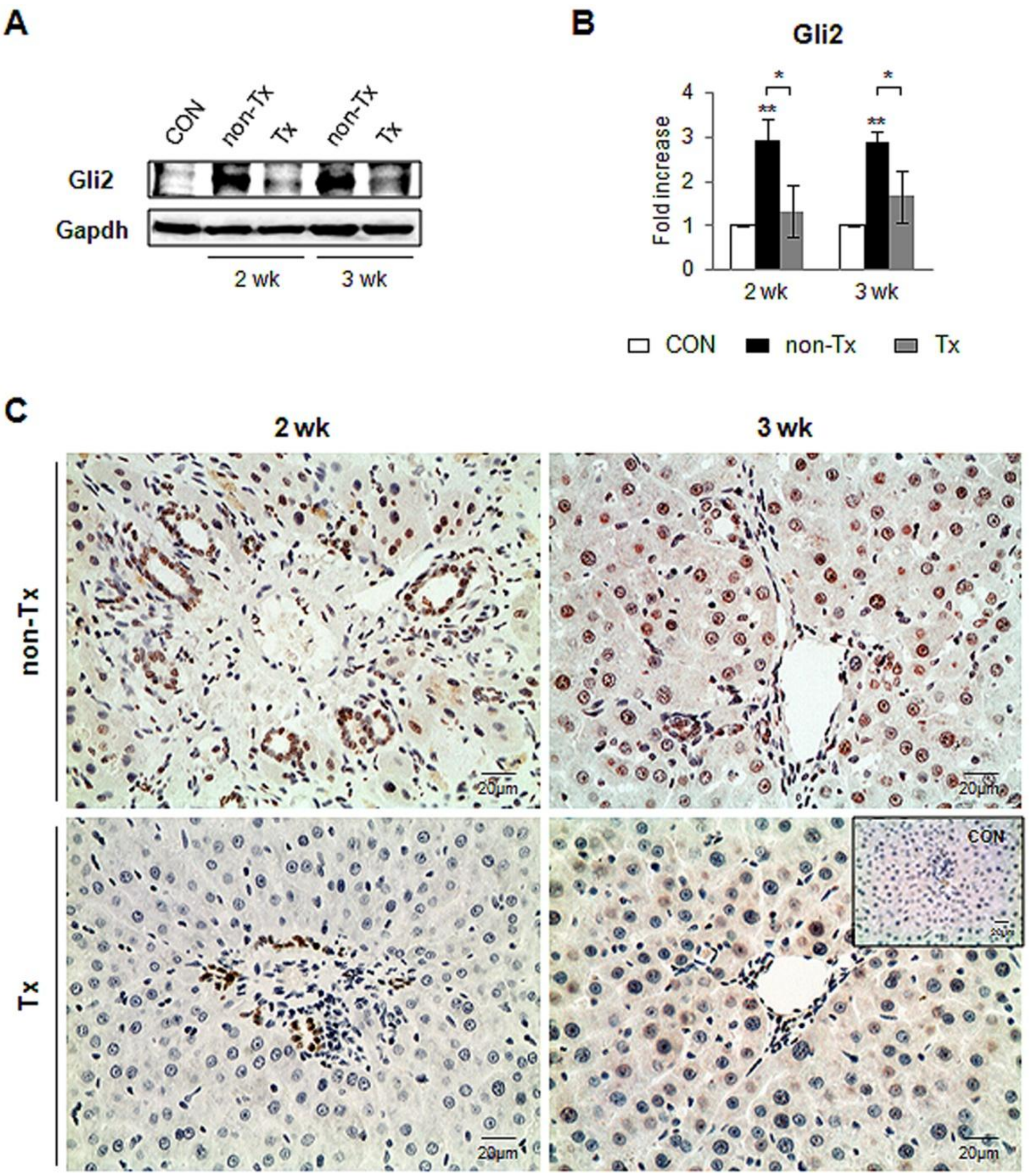

Supplementary Figure S2. Reduced expression of Gli2 in Tx rats. (A & B) Western blot analysis for Gli2 (133 kDa) and GAPDH (36 kDa, internal control) (n≥4 / group). Data shown represent one of three experiments with similar results (A: immunoblot/ B: band intensity). The mean±SD results obtained by measuring the band density of three different blots are graphed (*p<0.05, **p<0.005 vs. control). (C) Immunohistochemical staining for Gli2 in liver sections from the representative non-Tx and Tx rats at two and three weeks post transplantation (×40). The inserted image shows the representative liver section from corn oil-injected group (CON).

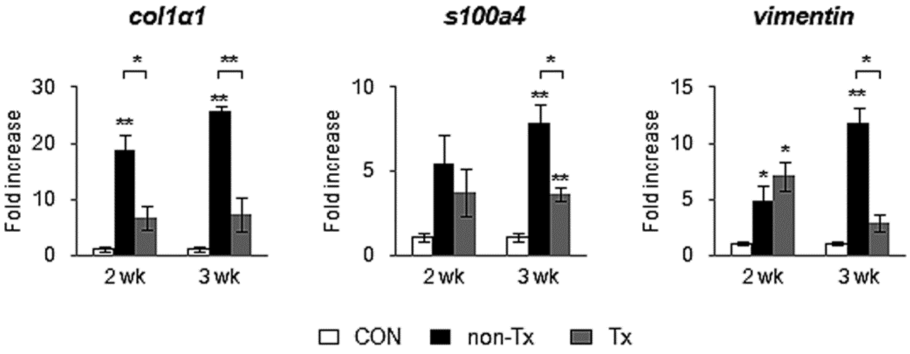

Supplementary Figure S3. Decreased expressions of EMT-related genes during liver regeneration. QRT-PCR analysis for EMT markers, including *col1α1, s100a4* and *vimentin*, in livers from the Tx, non-Tx and control (CON) rats (n≥4/group). The mean±SEM results are graphed (*p<0.05, **p<0.005 vs. CON).

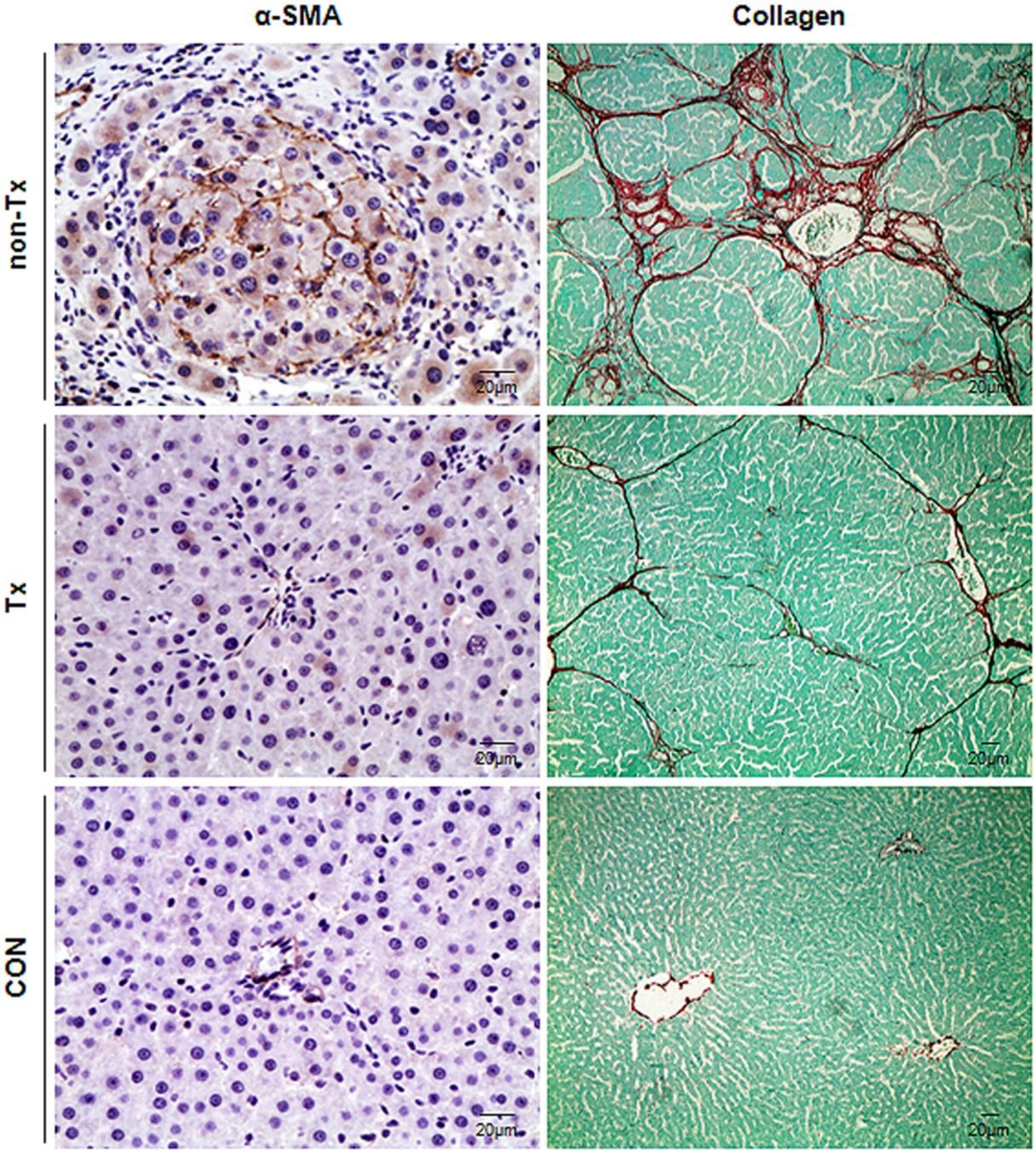

Supplementary Figure S4. Comparison of fibrosis in the Tx rats and Non-Tx rats at 3 weeks post transplantation. Staining results for α-SMA (×40) and sirius red staining (×10) were shown in liver sections from the representative non-Tx and Tx at 3 weeks post transplantation, and control (CON) rats.

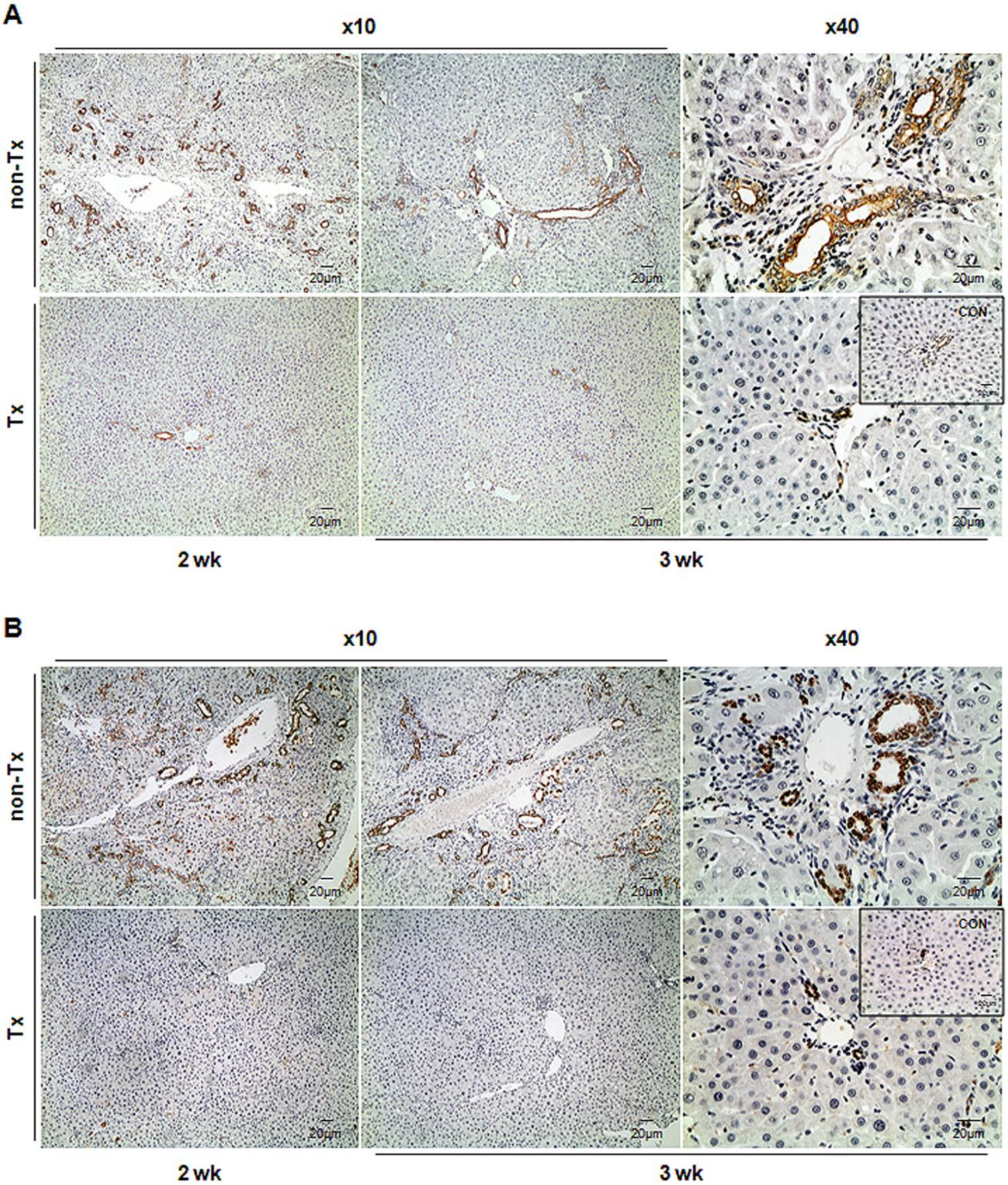

Supplementary Figure S5. Decreased accumulation of hepatic progenitors in liver from Tx rats. (A & B) Immunohistochemical staining for PanCK (A) and Sox9 (B), a marker of liver progenitor, in liver sections from the representative non-Tx and Tx rats at two weeks and three weeks after transplantation (×10 or ×40). The inserted images show the representative liver sections from corn oil-injected group (CON) (×40).

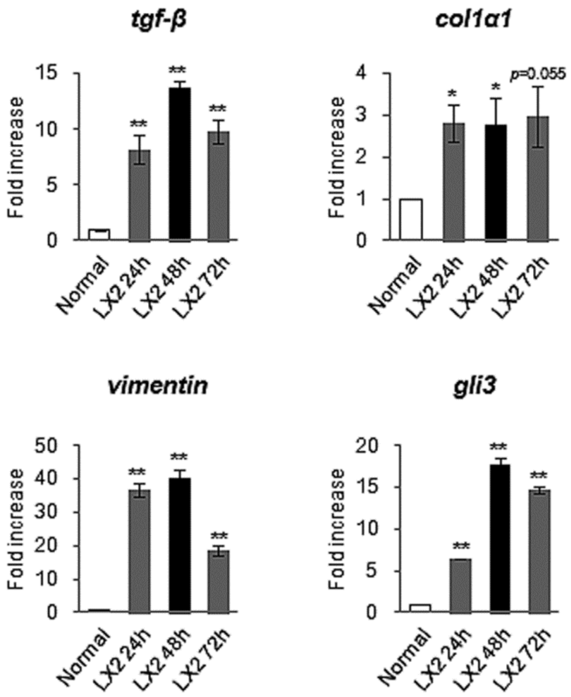

Supplementary Figure S6. Increased expression of Hh signaling and profibrotic genes in LX2 cell during culture. QRT-PCR analysis of expression of *tgf-β, col1α1, vimentin,* and *gli3* in the LX2 cells during culture for 24, 48, and 72 hours. The mean±SEM results obtained from three repetitive experiments are graphed (*$p<0.05$ vs. human normal liver).

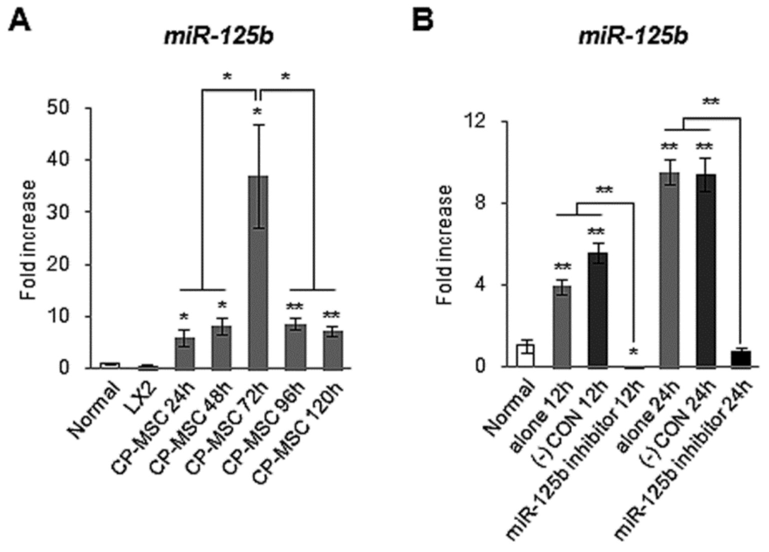

Supplementary Figure S7. The expressional changes of miRNA-125b in CP-MSCs with or without miRNA-125b inhibitor. (A) QRT-PCR analysis of the expression of miRNA-125b in human normal liver (Normal), LX2, and CP-MSCs cultured during 120 hours. The mean±SEM results obtained from three repetitive experiments are graphed (*p<0.05, **p<0.005 vs. Normal). (B) QRT-PCR analysis of miRNA-125b expression in human normal liver (Normal) and CP-MSCs transfected with or without miRNA-125b inhibitor (10nM) or scrambled-miRNA inhibitor (10nM) for 12 or 24 hours. Scrambled-miRNA inhibitor was used as a nagative control. The mean±SEM results obtained from three repetitive experiments are graphed (*p<0.05, **p<0.005 vs. Normal).

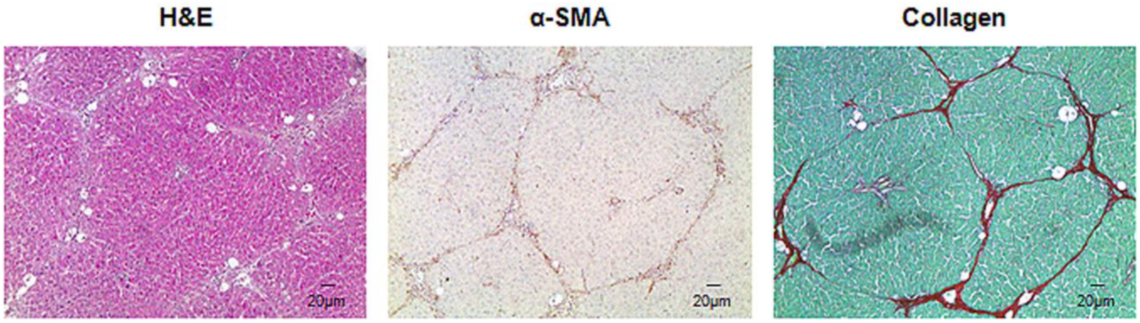

Supplementary Figure S8. Liver is chronically damaged by CCl_4 in rats. Representative images of H&E staining, immunostaining for α-SMA, and sirius red staining in liver sections from rats treated with CCl_4 for 5 weeks (×10).

Supplementary Table S1. Quantitative Real-Time PCR Primer Sequences of rat. Primer sequences shown in this table are used for QRT-PCR. rno-miR-125b was used in combination with miScript Universal primer (Qiagen). All values were normalized to the level of RPS9 or U1A snRNA for total mRNA or miRNA, respectively.

Gene	5' primer	3' primer
smo	AGAAGGCCTTGGCAATCAT	GAAGCCCATTCCTGATTGTG
gli3	CCCTCTCTCCCTTATCGTG	AAGGCAAGTCTGGATACGTT
tgf-β	TTGCCCTCTACAACCAACACAA	GGCTTGCGACCCACGTAGTA
α-sma	CGAGGTATCCTGACCCTGAA	GATGTGGTGCCAGATCTTCT
col1α1	GCCTCCCAGAACATCACCTA	GCAGGGACTTCTTGAGGTTG
s100a4	CTTCCGGGGCTCCTTATC	ATACTCAGGCAACGAGGGTG
vimentin	AATGCTTCTCTGGCACGTCT	CATCGTGCAGCTTCTTCAAA
shh	GGAACTCACCCCCAATTACA	TGCACCTCTGAGTCATCAGC
gli2	CAGTGGCAGTTGGTCTCGTA	ATAAGCGGAGCAAGGTCAAG
mmp9	AAATGTGGGTGTACACAGGC	TTCACCCGGTTGTGGAAACT
RPS9	GACTCCGGAACAAACGTGAGGT	CTTCATCTTGCCCTCGTCCA
[a]*rno*-miR-125b	TCCCTGAGACCCTAACTTGTGA	
U1A snRNA	CGACTGCATAATTTGTGGTAGTGG	

[a]rno; Rattus norvegicus (rat)

Supplementary Table S2. Quantitative Real-Time PCR Primer Sequences of human. Primer sequences shown in this table are used for QRT-PCR. hsa-miR-125b, hsa-miR-324-5p and hsa-miR-326 were used in combination with miScript Universal primer (Qiagen). All values were normalized to the level of RPS9 or U1A snRNA for total mRNA or miRNA, respectively.

Gene	5' primer	3' primer
smo	CTGGTGTGGTTTGGTTTGTG	AGAGAGGCTGGTAGGTGGTG
ptc	TCAGCAATGTCACAGCCTTC	ACTACTACCGCTGCCTGGAG
gli2	GTCTCCATCTCAGCCGCTCAT	TCCGCCGTCTAGTGAAGTTCGT
gli3	GGTGTTTGGCGCGATCAG	GAAGACACACGGGCGAGAAG
tgf-β	TTGACTGAGTTGCGATAATGTT	GGGAAATTGCTCGACGAT
col1α1	CAGATCACGTCATCGCACAA	TGTGAGGCCACGCATGAG
vimentin	CGAAAACACCCTGCAATCTT	GTGAGGTCAGGCTTGGAAAC
mmp9	CGAACTTTGACAGCGACAAG	GCCATTCACGTCGTCCTTAT
shh	GATGTCTGCTGCTAGTCCTCG	AGAAGCAGGGTCAAGAGTGGTGAA
RPS9	GACTCCGGAACAAACGTGAGGT	CTTCATCTTGCCCTCGTCCA
[a]hsa-miR-125b	TCCCTGAGACCCTAACTTGTGA	
hsa-miR-324-5p	CGCATCCCCTAGGGCATTGGTGT	
hsa-miR-326	CCTCTGGGCCCTTCCTCCAG	
U1A snRNA	CGACTGCATAATTTGTGGTAGTGG	

[a]hsa; Homo sapiens (human)

Supplementary Methods

RNA analysis

Total RNA was extracted from liver tissues by using easy-BLUE™ Total RNA Extraction Kit (iNtRON Biotechnology) and TRIzol Reagent (Ambion, Life technologies) or miRNeasy Mini Kit (Qiagen) to enrich miRNA quantity. The concentration and purity of RNA were determined using a nanodrop (Thermo Scientific, Waltham, MA). Template cDNA was synthesized from total RNA using the SuperScript First-strand Synthesis System (Invitrogen, Life technologies) and SuperScript VILO Master Mix (Invitrogen, Life technologies) or miScript Reverse Transcriptase Kit (Qiagen) according to the manufacturer's protocols. We performed the quantitative reverse transcription polymerase chain reaction (QRT-PCR) by using Power SYBR Green Master Mix (Applied Biosystem, Life technologies) or miScript SYBR Green PCR Kit (Qiagen) on the manufacturer's specifications (Eppendorf, Mastercycler Real-Time PCR). All reactions were duplicated and data were analyzed according to the $\Delta\Delta C_t$ method. 40S ribosomal protein S9 (RPS9) mRNA or U1A small nuclear RNA (RNU1A) was used to normalization of the expression level of mRNA or miRNA, respectively. The sequences of all primers used in this study are summarized in Supplementary Table S1 and S2.

Western blot analysis

Frozen liver tissues stored at -80℃ were homogenized in triton lysis buffer (TLB) and centrifuged at 13,000 rcf for 15 minutes. The supernatants containing protein extract were used in subsequent biochemical analysis. Protein concentration was measured by Pierce BCA Protein Assay Kit (Thermo Fisher Scientific Inc.). To quantify the expression level of Hh signaling molecules and fibrosis markers, we performed western blot analysis. Total protein

lysates were pooled from individuals (n = 4) of equal concentration, separated by 10 % SDS-PAGE and then transferred onto a PVDF membrane (Millipore Corp.). Primary antibodies used in this study were as follows: Mouse anti-α-SMA antibody (diluted 1:1,000; Sigma-Aldrich), rabbit anti-TGF-β antibody (diluted 1:1,000; Cell Signaling Technology, Inc.), rabbit anti-Shh antibody (diluted 1:5,000; Epitomics, Inc.), rabbit anti-Ihh antibody (diluted 1:1,000; Abcam), rabbit anti-Smoothened antibody (diluted 1:1,000; Abcam), rabbit anti-Gli2 antibody (diluted 1:1,000; GenWay Biotech, Inc.), rabbit anti-Gli3 antibody (diluted 1:1000; Abcam), rabbit anti-CD-9 antibody (diluted 1:1,000; Abcam), and mouse anti-GAPDH antibody (diluted 1:1,000; AbD Serotec.) as an internal control. Horseradish peroxidase (HRP)-conjugated anti-rabbit or anti-mouse IgG (Amersham ECL™, GE Healthcare) was used as secondary antibody. Protein bands were detected using an EzWestLumi ECL solution (ATTO Corporation) per the manufacturer's specifications (ATTO Corporation, Ez-Capture II). Densities of protein bands were measured using CS Analyzer software (Version 3.00.1011, ATTO & Rise Corporation).

Hydroxyproline assay

Hydroxyproline content of the livers was calculated by the method previously described.[19] Briefly, 50 mg of freeze-dried liver tissue was hydrolyzed in 6N HCL at 110 ℃ for 16 hours. The hydrolysate was evaporated under vacuum and the sediment was re-dissolved in 1 ml distilled water. Samples were filtered using 0.22 ㎛ filter centrifuge tube at 14000 rpm for 5 minutes. Lysates were then incubated with 0.5 ml of chloramines-T solution, containing 1.41 g of chloramine-T dissolved in 80 ml of acetate-citrate buffer and 20 ml of 50% isopropanol, at room temperature (RT). After 20 minutes, 0.5 ml of Ehrlich's solution, containing 7.5 g of dimethylaminobenzaldehyde dissolved in 13 ml of 60% perchloric acid and 30 ml of isopropanol, was added to the mixture, which was incubated at 65℃ for 15 minutes. After cooling to the RT,

the absorbance was read at 561 nm. Hydroxyproline concentration was calculated from a standard curve prepared with high purity hydroxyproline (Sigma-Aldrich) and divided by the weight of liver specimen which was employed in this analysis (mg hydroxyproline/g liver). Data were expressed as fold changes by comparing with hydroxyproline content of the control group.

Immunohistochemical staining

Collected liver specimens were fixed in 10 % buffered formaldehyde, embedded in paraffin and cut into 3 ㎛ sections. For immunohistochemistry (IHC), specimens were deparaffinized, hydrated and incubated in 3 % hydrogen peroxide to block endogenous peroxidase. Antigen retrieval was performed by heating in 10 mM sodium citrate buffer (pH 6.0) for 10 minutes using microwave or applying the pepsin (Sigma-Aldrich) for 10 minutes at 37 ℃. Specimens were blocked in Protein Block solution (Dako) for 30 minutes and incubated with primary antibody at 4 ℃ overnight. Other sections were also incubated at 4 ℃ overnight in non-immune sera. Primary antibodies for IHC were as follows: Rabbit α-SMA antibody (diluted 1:500; Abcam), rabbit anti-Shh antibody (diluted 1:300; Santa Cruz Biotechnology, Inc.), rabbit anti-Ihh antibody (diluted 1:1,500; Abcam), rabbit anti-Gli2 antibody (diluted 1:2000; GenWay Biotech, Inc.), rabbit anti-Gli3 antibody (diluted 1:250; Abcam), rabbit anti-cytokeratin antibody (diluted 1:2,000; Dako), and rabbit anti-Sox9 antibody (diluted 1:2000; Millipore). All primary antibodies were diluted in Protein Diluent (Dako), and polymer-HRP anti-rabbit (Dako) was used as secondary antibody. Proteins were visualized by 3,3'-diaminobenzidine (DAB) as brown color.
